# Multi-Hazard Effects of Crosswinds on Cascading Failures of Conventional and Interspersed Railway Tracks Exposed to Ballast Washaway and Moving Train Loads

**DOI:** 10.3390/s23041786

**Published:** 2023-02-05

**Authors:** Hao Fu, Yushi Yang, Sakdirat Kaewunruen

**Affiliations:** Laboratory for Track Engineering and Operations for Future Uncertainties (TOFU Lab), Department of Civil Engineering, School of Engineering, The University of Birmingham, Edgbaston, Birmingham B15 2TT, UK

**Keywords:** vulnerability, railway, interspersed tracks, finite element method, extreme conditions, multi hazards, cascading failure, resilience

## Abstract

The interspersed railway track is an enhanced timber railway track, spot-replacing damaged wooden sleepers with new concrete sleepers to improve the bearing capacity of existing railway lines. Although this interspersed solution is characterised by low cost and short maintenance time, the interspersed tracks have worse stability than concrete tracks and can deteriorate quickly when exposed to extreme weather conditions such as heavy rains and floods. In many cases, heavy rains and floods are accompanied by strong winds. Ballast washaway can often be observed under flood conditions while the mass of trains is unevenly distributed on two rails due to the effect of lateral wind load and rail irregularities. The current work is the first in the world to investigate the collective multi-hazard effects of ballast washway and uneven axle loads on the vulnerability of conventional and interspersed railway tracks using nonlinear FEM software, STRAND 7. The train bogie is modelled by two sets of point loads. The maximum displacement, bending moment and twists have been studied to evaluate the worst condition. The novel insights will help the railway industry develop proper operations of interspersed railway tracks against naturally hazardous conditions.

## 1. Introduction

**Figure 1:**
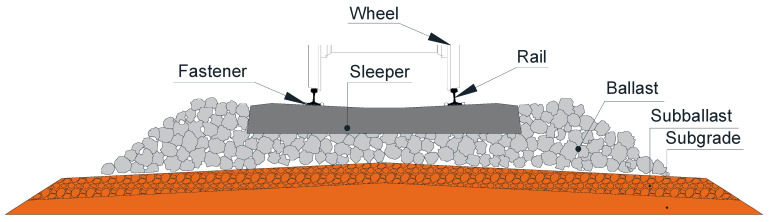
Components of typical ballasted railway track.

According to the support layer type, railway transportation systems can be classified into ballasted track systems and concrete slab track systems. The ballasted railway systems have been adopted globally because of low construction cost, short maintenance interval and good elasticity in the last two centuries [1]. Almost all normal-speed railway lines and heavy-haul lines adopted the ballasted track system. The ballasted railways have also been proven applicable for high-speed railway lines such as French TGV lines, German ICE lines and Spanish NAFA lines [2,3]. Ballasted railway system usually comprise rails, fasteners, rail pads, sleepers, a ballasted bed, a sub-ballasted layer and a subgrade, as shown in Figure 1. The sleepers are critical for supporting the rails, redistributing the axle force and keeping the rail gauge. Railway sleepers can be divided into timber, concrete, composite and steel sleepers according to the material type [4,5,6]. Timber sleepers had been widely used before the mid-1950s due to vast resources and low cost. Nevertheless, timber sleepers are prone to degradation under long-term service as a consequence of poor resistance to chemical and biological reactions and low bending capacity [6]. On many existing timber railway lines, the timber sleepers have been observed cracking and ageing, resulting in insufficient track stiffness and inappropriate rails’ position. The lifecycle of timber sleepers is around 15–20 years. Degraded timber railway lines are required to be enhanced to meet the safety and comfort requirements of the railway transportation systems [7,8].

**Figure 2:**
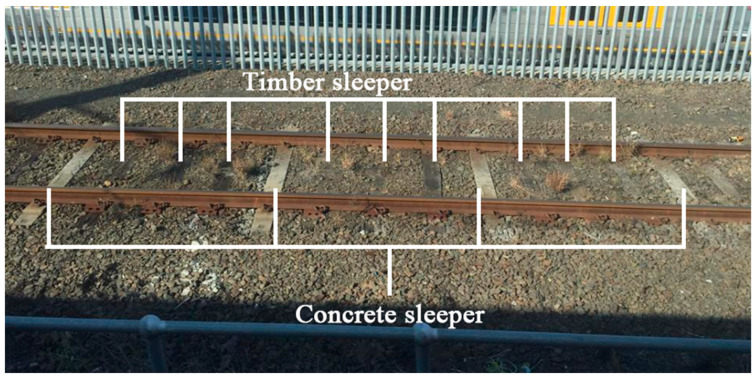
A 1in4 interspersed ballast track [12].

Regular maintenance and replacement of sleepers at the existing railways are the simplest and most widely used way to repair timber railway track systems [9,10]. Prestressed concrete sleepers have been widely used as railway sleepers, especially for high-speed railway lines. Their economic and technical advantages are their long service life and high resistance against physical and environmental reactions. Concrete sleepers can provide the rails with optimum position persistence and stability by their vast weights. Concrete sleepers can provide higher track stiffness and have better environmental resistance than timber sleepers, and have become the most preferred option for building new ballasted railway lines [1]. Replacement of timber sleepers with concrete sleepers on site is a method for improving the performance of the existing timber lines. However, full sleeper replacement is not cost-effective, considering the overall performance and the charge of construction work [9,10]. The compromise approach, partial replacement of timber sleepers on an existing timber railway line, is feasible. A timber ballasted track where concrete sleepers have replaced some timber sleepers is an interspersed railway track. This interspersed approach can improve track quality, such as track stiffness, lateral resistance and longitudinal resistance, in a short operating time to prevent track buckling [11,12,13,14,15,16]. The interspersed method has been proven to have sufficient track quality and has been adopted in Australia, Japan, England and America [5,17]. The placement density of concrete sleepers on an interspersed ballasted track impacts the track performance [15,16]. The interspersed track with a high concrete sleeper placement density deforms less and has a lower bending moment at midspan than a low concrete sleeper placement density interspersed track. The most common placement density of concrete sleepers on an interspersed ballasted track is 1in4 (spot-replacement of a timber sleeper by a concrete sleeper every four sleepers), as shown in Figure 2. Interspersed railway tracks with higher concrete sleeper density (1in2 and 1in3) have also been utilised in some railway networks with slightly higher speeds than normal-speed railway lines [18].

**Figure 3:**
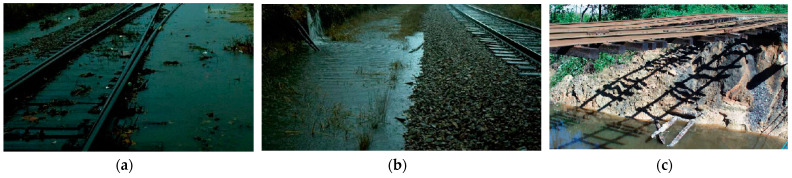
Typical track conditions caused by flood or heavy rain: (**a**) ballast drawn in water,(**b**) subgrade drawn in water, (**c**) fully unsupported sleepers caused by ballast and subgrade washing away.

Natural disasters are common challenges faced by railway transportation systems. Most railway infrastructures, except metro lines, were constructed in an open environment with a relatively higher possibility of being affected by natural disasters such as heavy rains, floods and mudslides compared with civil architecture in human settlements [19,20]. Besides, periodic typhoons, hurricanes and storms have also caused increasing concerns about the risk and safety issues of the railway systems in certain areas. For example, the south-east Asian regions, including Thailand, Malaysia, etc., have two dominating periodic monsoon regimes [21]. These can result in heavy precipitation in some districts in a short period and even cause regional flooding. These extreme weather conditions may cause the ballasted track infrastructures to be drawn. Similar railway flood issues were observed in China [22]. The water inside ballasted track can cause subgrade softening and ballast washing away, or unsupported sleepers under moving train loads. Some typical track conditions caused by floods and ballast washing away are displayed in Figure 3a–c. The cascading damage to ballasted railway support layers may cause uneven settlement of sleepers, leading to vehicle body inclination. In severe cases, it will result in train rollover and derailment, endangering life safety and inducing adverse social and economic effects [22,23]. Therefore, it is necessary to study the vulnerability of the interspersed railway tracks under heavy rain or flooding conditions. Understanding the factors affecting the multi-hazard vulnerability of railway tracks can help reduce loss and prevent severe incidents from occurring in advance.

Previous studies have shown that the interspersed method is more economical than fully replacing aged timber sleepers [9]. The interspersed railway systems have enough track quality to operate with a considerable train speed under normal weather conditions [13,14,15,16,17,24]. Nevertheless, the approach would result in inconsistent track stiffness due to the elasticity difference between the renewed concrete sleepers and the unreplaced aged timber sleepers [15,25]. The track stiffness inconsistency is a reason for uneven settlement and foundation failure in later operation and can impair the ballast and subgrade layers. Besides, it has been pointed out by [16] that the interspersed track should be operated under 20 km/h with vigilant monitoring and control when suspected to be suffering from ballast washing away. According to previous studies on interspersed railway tracks, interspersed tracks have greater dynamic twists than concrete tracks and are prone to deteriorate. Notably, the interspersed track has poor resistance against water infiltration. Moreover, interspersed tracks should operate under a limited speed when exposed to ballast washaway conditions. However, previous studies of the vulnerability to interspersed tracks exposed to heavy rain (flood) conditions have not considered the unequal axle loads caused by crosswind load and rail-wheel defects. In reality, heavy rain weather conditions or flood conditions are often accompanied by strong winds; also, the difference in rail and wheel corrugation on two rails can induce different dynamic moving loads on two rails [26]. The unevenly distributed axle loads can result in uneven rail settlement, endangering ride safety. It is necessary to evaluate the combined cascading effects of the ballast washaway and the uneven axle loads.

Thus, this research carried out three-dimensional finite element modelling of interspersed railway tracks under different train speeds exposed to ballast washing away and uneven axle load conditions using Strand 7. Dynamic responses, including rail twists and sleeper bending moments under moving train loads, have been compared to assess the serviceability level facing multi hazard conditions. The new insights into interspersed ballasted railways will help railway engineers adopt proper measures for conventional and interspersed ballasted tracks under heavy rain, flood or storm weather conditions.

## 2. Methodology

### 2.1. Track Modelling

**Figure 4:**
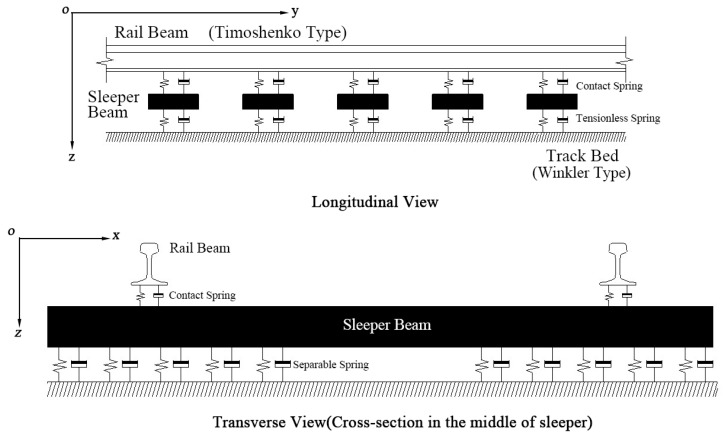
1:4 Idealized rail track model.

Conventional FEM models of a ballasted track system considering the moving train loads adopted the Bernoulli-Euler type beam to simulate the rails sitting on Winkler foundation, representing the combined stiffness of the track supporting components: rail pads, sleepers and ballasted bed. However, this approach cannot investigate the performance of discrete railway parts and the bending behaviour of the sleepers. In the literature, the application of Timoshenko beam theory to simulate both the rails and sleepers as flexible beams can take into account shear and flexural deformation [27,28,29,30]. However, there is still a limitation of many FEM models in reflecting the practical ballast–sleeper contact interface, where the sleeper and ballast can be separated when railway ties are lifted by the rails. In most research, the sleeper and ballasted supporting are bonded or non-separable. This paper aims to compare the dynamic performance of interspersed, concrete, and timber tracks under the ballasted washing away conditions and crosswind effects. In order to reflect the real ballast sleeper interactions, tensionless beam supports of sleepers are adopted [31,32]. The Timoshenko beam is employed to model rails and all types of sleepers. The contact spring is utilised to represent the resilient fastener systems. The idealized track system is present in Figure 4.

**Figure 5:**
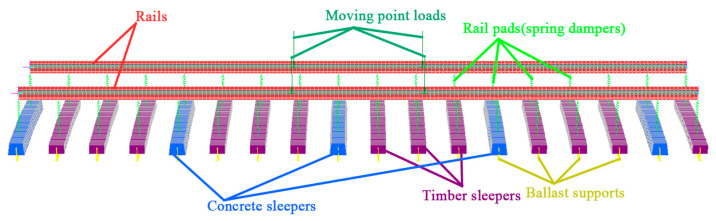
1:4 interspersed track FEM model.

The three-dimensional ballasted track models with standard gauges are built using commercial software Strand7, as presented by [16]. Model validations were carried out using experimental and laboratory results [13,14,15,16,33]. The entire model comprises rails, rail pads, sleepers and ballast supports. The rails and concrete sleepers are simulated using the Timoshenko beams, which take the shear and flexural deformations into consideration [12]. The rail beams are modelled by 200 beam elements with UIC60 steel rail geometry properties (A = 76.70 cm^2^, Mass = 80.21 kg/m, Ixx = 3038.3 cm^4^ and Iyy = 512.3 cm^4^) [25]. The cross-section of the timber sleepers and the concrete sleepers are 230 mm wide × 130 mm deep and 204 mm top-wide × 250 mm bottom-wide × 180 mm deep, relatively, according to [12,25]. Each sleeper is modelled using 60 beam elements. The sizes of the beam elements for sleepers and rails are determined after a mesh independence study. The railway fastener systems at rail-seat are modelled using a series of spring dampers with the stiffness and damping values of high-density polyethene pads based on experimental data [34]. The ballast layer is modelled as a series of compression-only elastic supports. These supports allow the sleepers to be lifted up as the tensile stiffness of the supports is set to 0 when sleeper lift happens. The compression-only support can simulate the real ballast-sleeper interaction properly [15,16]. This research has adopted three types of widely utilised interspersed railway tracks, one timber track and one concrete track. The interspersed tracks are 1in2, 1in3 and 1in4 interspersed tracks. The 1in2 interspersed track involves a particular arrangement of timber and concrete sleepers, where one concrete sleeper and one timber sleeper are adopted in every two sleepers along the railway line. Similarly, 1in3 and 1in4 interspersed tracks indicate that one concrete sleeper is located next to every two and three timber sleepers along the railway track along the railway line. An example of the 1in4 interspersed railway FEM model is displayed in Figure 5.

### 2.2. Risk Exposures to Flooding and Heavy Rain Conditions

#### 2.2.1. Sleeper Support Conditions

**Figure 6:**
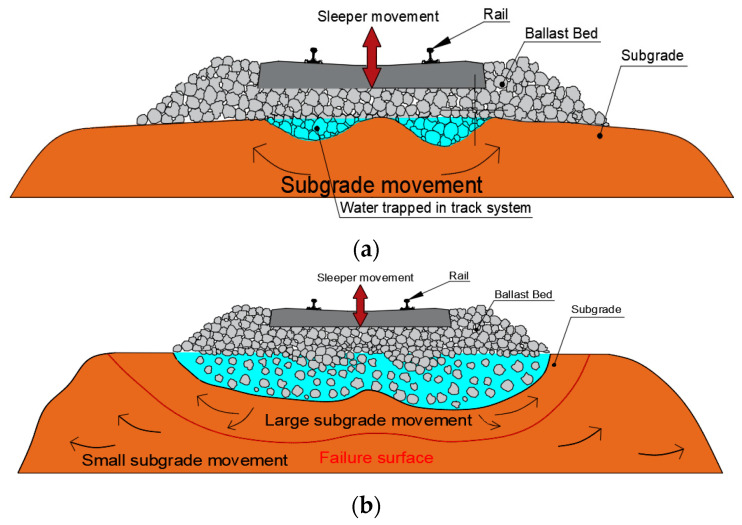
Deterioration of ballasted track under water ponding conditions: (**a**) the softening and movement of subgrade layer and (**b**) the ballast particle rearrangement.

The support infrastructures of an interspersed ballasted track are composed of a ballasted bed, sub-ballast layer and subgrade. The three layers are assembled of discrete crushed rocks and soil bulk materials. When the infrastructures are exposed to soaking or flood conditions, the subgrade may be softened, moved or washed away under the dynamic moving train loads [16]. The mechanical properties of the track will deteriorate. The bottom infrastructures under the sleeper deteriorate when subject to moving train load and water ponding conditions. As shown in Figure 6a, the liquefaction of soil bulk near the ballast-subgrade interface occurs under the seismic vibration caused by the dynamic moving train loads. The soil particles start to move towards both sides and vertically under the axle loads, and the liquefaction area develops much more broadly, as presented in Figure 6b. The ballast bed layer becomes loose. The crushed ballasted graded gravels move into the subgrade layer. The sleeper support conditions may change after the combined effects of the water ponding and dynamic trainloads and differ according to the severity of the damage and the deterioration inside the ballast bed and subgrade layer [35,36,37,38,39,40].

**Figure 7:**
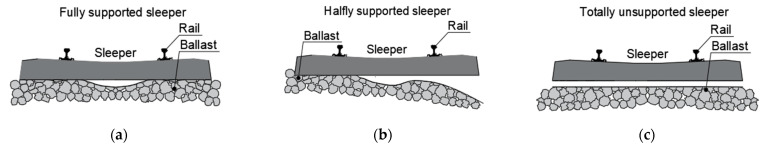
Different sleeper support conditions: (**a**) fully supported sleeper, (**b**) half supported sleeper and (**c**) totally unsupported sleeper.

**Figure 8:**
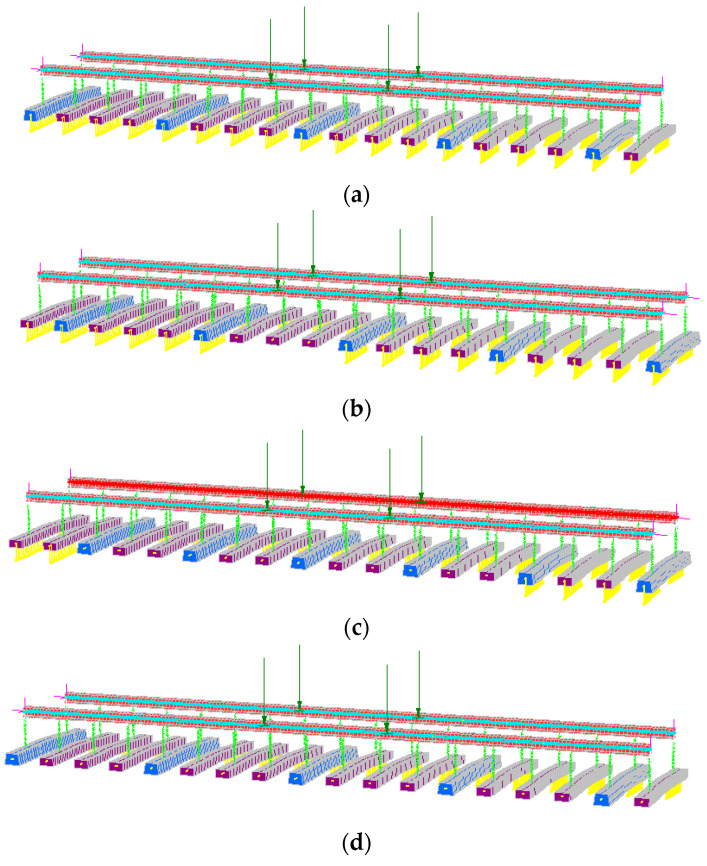
Different track support conditions under even axle loads: (**a**) normal support condition, (**b**) small-scale loss support condition, (**c**) large-scale loss support condition and (**d**) full-scale loss support condition.

Three typical sleeper support conditions are present in Figure 7 [16,19]. Initially, the track supporting stiffness will decrease when damage to the support layers occurs. When the condition becomes severe, the half sleeper support condition (Figure 7b) may occur. In most cases, the half supported sleeper condition cannot be observed because the ballasted track’s overall shape does not change much. The fully unsupported sleeper condition (Figure 7c) only occurs when the flood is severe, or mudslides happen. When complete loss of support occurs, the ballast bed under the sleeper has been thoroughly washed away, or the hanging sleeper can be noticed. The railway lines will be stopped. Therefore, the half-support condition of the sleeper was adopted for this research to simulate the ballast washing away effect, considering reasonable operating conditions. According to the intensity difference of flood or heavy rainfall, four support states are designed: full support, small-scale loss, large-scale loss and full-scale loss, as shown in Figure 8.

#### 2.2.2. Crosswind Effects

**Figure 9:**
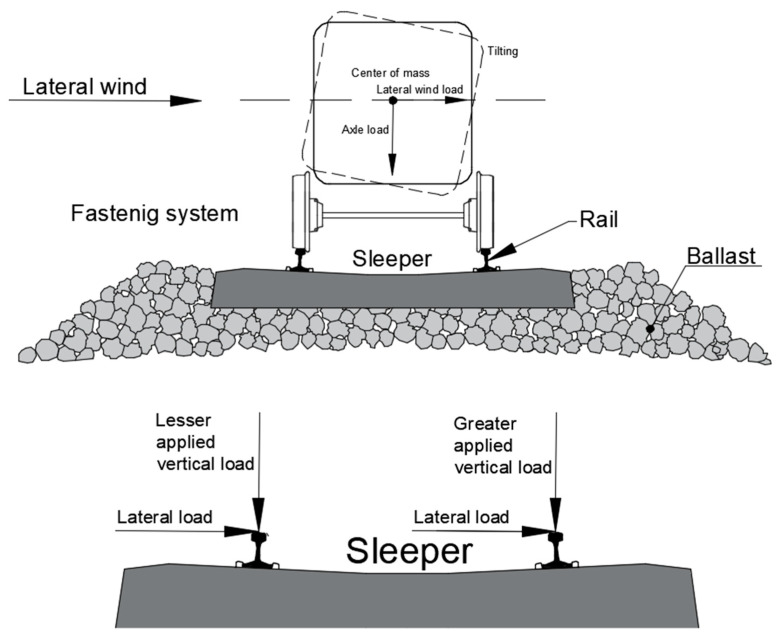
Force transfer of the railway under later wind loads.

In most cases, heavy rainfall weather conditions do not happen alone. Strong winds often accompany rainstorm weather conditions. Strong crosswinds may further endanger the safety of railway train operations and even lead to the overturning and derailment of a train in extreme cases, as stated by [41,42]. As a result, the speed of trains needs to be diminished to ensure a safe run under conditions with floods and strong winds. In order to evaluate the overturning risk of running vehicles under the effect of transverse wind, it is first necessary to calculate the track response. Currently, the index used to evaluate the overturning accident of a train is the wheel load reduction rate [43,44,45]. Figure 9 shows the force induced by lateral wind loads at the rail–wheel contact interface. Note that loading from sources other than static and wind is not listed in the diagram. The lateral wind force acts at the mass centre of the train. Therefore, a specific moment is taking effect on the track system, causing the train body to tilt in addition to a purely lateral force. At the same time, the moment manifests itself in terms of uneven force distribution on the two rails. At operating railway lines, the difference between the axle loads on two rails under lateral wind load is much more significant due to the dynamic loads caused by irregularities and the track stiffness discontinuity.

To recognize the crosswind influence on train–track dynamics, the research on characteristics of crosswinds and the aerodynamic wheel–rail contact forces based on experimental tests and numerical theories has been studied in depth [46,47,48,49,50,51,52]. According to BS EN 14067-6:2010, a crosswind will induce side force, lift force and roll moment to a car body [53]. The wind loads on vehicles are determined by the wind velocity relative to the vehicle, the side area of the car body, the density of air and the relative aerodynamic coefficients [48,52]. In practical railway operations, the train speed will be limited according to the wind speed. For example, the train should run under the speed limitation of 60 km/h for the seventh wind level and 25 km/h for the eighth wind level. Railways should shut down when the wind level is stronger than the ninth level in China. Thus, the wind speed of 17.2–20.7 m/s corresponding to the eighth wind level is adopted. According to [46,52,54], the crosswind at the eighth wind level and the imperfect rail–wheel contacts will result in 50% changes in wheel–rail vertical contact force. Therefore, in this study, 50% of the axial force and 150% of the axial force are applied on two rails to reflect the effect of crosswind under the seventh wind level for the most dangerous situation.

In order to study the combined effects of the heavy rain (flooding) and lateral wind load on the interspersed ballasted track, the half supported sleeper condition and the uneven axle loads on two rails are adopted. The half supported sleeper condition can reflect the invisible onsite track supporting damage in operating lines under ballast washing away and softened subgrade states. The small loss, large loss and full loss of sleeper supports can reflect the severity of damage to the track infrastructures by heavy rain (floods). The uneven axle loads, 50 kN and 150 kN on two rails, are adopted to consider the lateral wind load and the practical dynamic moving train loads. The greater axle load is applied on the rail surface on the unsupported side to consider the most critical situation. It was suggested by [13,16] that the interspersed track should operate under 60 km/h. Thus, the dynamic responses of different interspersed tracks, timber and concrete tracks are analysed under speeds of 20 km/h, 40 km/h and 60 km/h to compare with the Australian TMC 203 track inspection [55].

### 2.3. Track Modelling

The material properties and the geometric parameters of the track models are presented in Table 1. The material properties have been determined and validated from past research [13,14,15,16,33]. The sleeper spacing is 0.6 m. To consider the ballast washing away effects, the half support of sleepers is adopted. The trainloads are simulated using a group of moving point loads. Two point loads with the same magnitude of 100 kN and a distance of 2 m on each rail are used to reflect the axle loads from a passenger train bogie. According to [46,52], the magnitudes of 50 kN and 150 kN of the axle loads on different rails are utilised to study the combined effect of losing support caused by ballast washing away and uneven axle loads caused by wind load and rail wear. It should be noted that loads of 150 kN are applied on the unsupported side for consideration of the worst-case condition. The load distribution and magnitudes are used as a benchmark. The dynamic twists of the track models are compared with the Australian Railway Operating Manual [55]. The sleeper bending moments are analysed.

## 3. Results and Discussion

### 3.1. Track Responses

**Figure 10:**
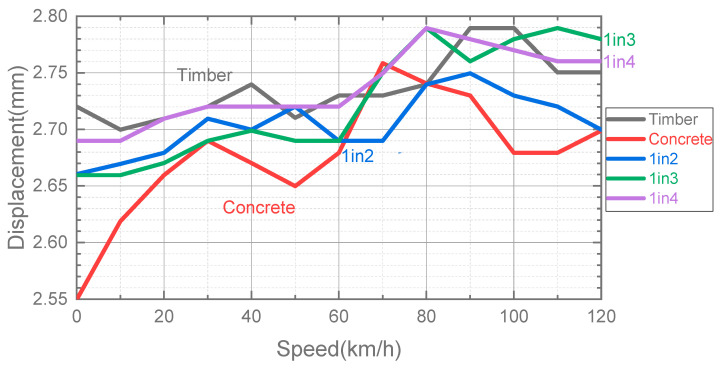
Maximum rail displacement of different fully supported tracks under different train speeds.

Figure 10 presents the maximum vertical rail displacement of different interspersed tracks, a timber track and a concrete track under static load and moving train loads with 10 km/h to 120 km/h speed. The dynamic rail displacement of all types of tracks tends to increase as the train speed increases. Generally, the concrete track has the smallest rail displacement, while the timber sleeper has the greatest displacement at the same train speed. The maximum displacement values of interspersed tracks are between concrete and timber sleeper. This is because concrete sleepers can provide larger track stiffness than timber sleepers, accounting for different dynamic track responses against excitation frequencies. The symmetric dynamic responses can be observed because the fully supported track models have symmetric geometric properties, constraints and input moving loads. The symmetric dynamic responses (e.g., same displacement on two rails) would affect the ride comfort and may induce higher dynamic force at the wheel–rail interface, resulting in rail and wheel corrugations and louder noises [56].

**Figure 11:**
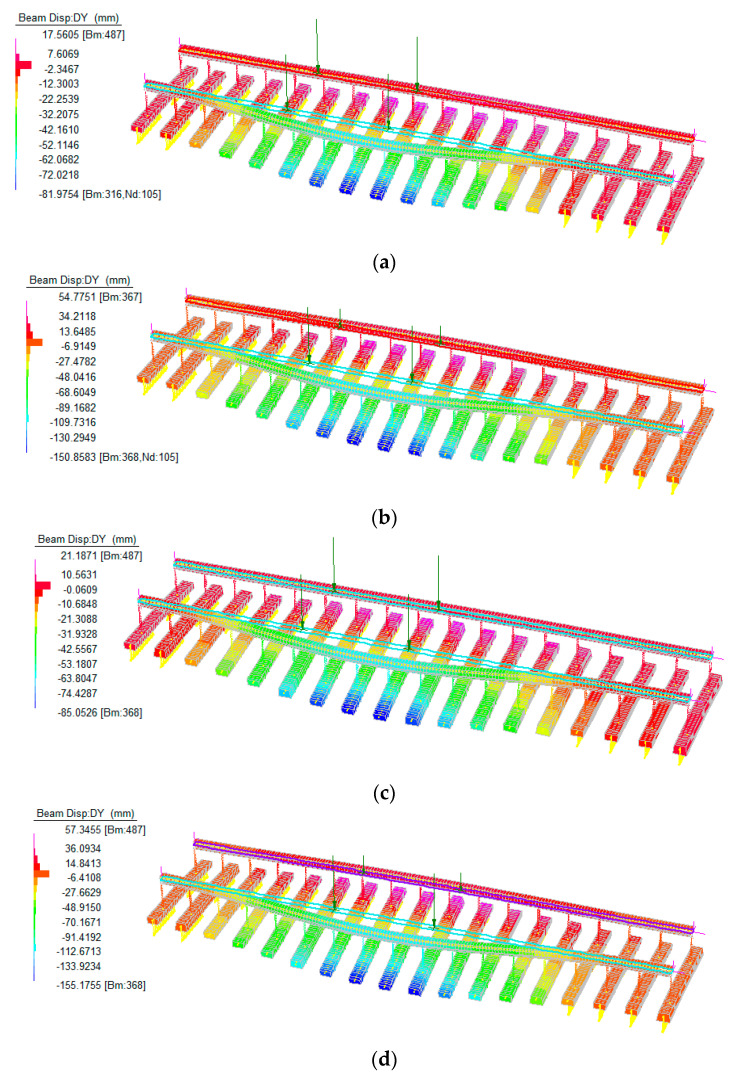
Dynamic track response under different track support states and axle loads at 60 km/h. (**a**) timber track with even axle loads and largescale loss support, (**b**) timber track with uneven axle loads and large–scale loss support, (**c**) 1in4 track with even axle loads and large–scale loss support and (**d**) 1in4 track with uneven axle loads and large–scale loss support.

To compare the vulnerability of 1in4 interspersed track and timber tracks under heavy rain (floods) and strong wind conditions, the maximum dynamic responses of these tracks exposed to large-scale loss of sleeper support at 60 km/h are presented in Figure 11. The half sleeper support condition and uneven axle loads are adopted to reflect the ballast washaway condition caused by heavy rain (flood) and the lateral wind condition. It can be seen from Figure 11 that the interspersed tracks have better track performance than timber tracks under the same load and track support conditions. The uneven group load will induce higher deformation in track sleepers. Besides, the moving loads and the poor track support state can result in unequal vertical rail displacement on two rails. The height difference between two rails is usually referred to as ‘cross level’. This elevation difference may result in train derailments. In order to keep the rail lines in proper operating condition, short twists and long twists are often used to judge the rail elevation conditions. If either twist value exceeds the suggested value, certain track measures and repairs must be carried out to improve the track status.

The short and long twist limits are listed in Table 2 according to the Australian railway maintenance manual [55]. The twist limit values for low-speed railway lines are higher than high-speed lines. The defects are categorised into six standard defect categories: N (Normal), P3 (Priority 3), P2 (Priority 2), P1 (Priority 1), E2 (Emergency 2) and E1 (Emergency 1). For each defect condition, the repair action and track response inspection should be executed within a limited period, as presented in Table 3. If the track is under E conditions (E1/E2), the running train is prone to derail and the track system needs to be maintained for better track quality before the next train operation.

**Figure 12:**
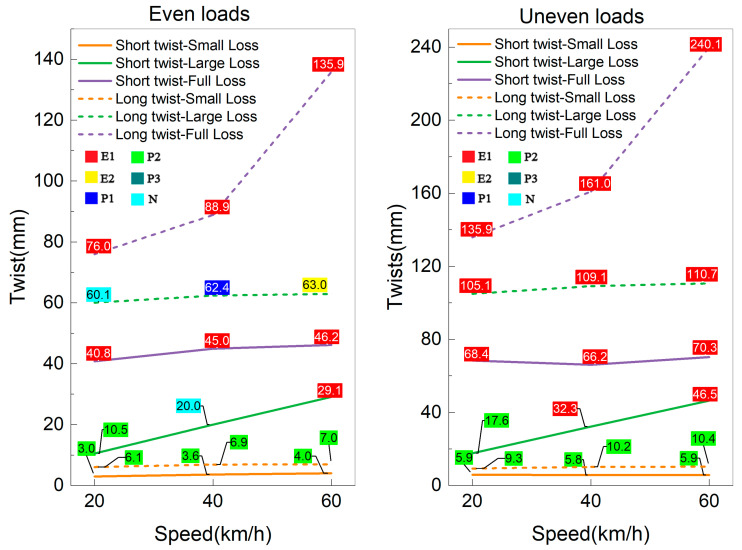
Dynamic twists of 1in4 interspersed tracks under different track support and load conditions.

Figure 12 presents the dynamic twists of the 1in4 interspersed track considering the small-scale, large-scale and full-scale support loss conditions under even and uneven moving loads. The track twist conditions have been coloured based on the risk level according to the defect categories in Table 2. The result implies that the uneven group loads can induce higher dynamic twists than even loads. The 1:4 interspersed track exposed to small-scale and large-scale support loss conditions is at risk of derailment when the train speed exceeds 40 km/h. The railway line should be stopped and repaired immediately if the track support state is a full-scale loss. From this aspect, operators should take more care when the track is exposed to heavy rain (flood) and intense wind. Some measures can be adopted to improve the sleeper support state, such as spraying ballast glue and utilising geogrids. This finding will help railway practitioners to adopt proper actions towards climate change and suitable operations of interspersed ballasted tracks against heavy rain (floods) and strong wind conditions.

**Figure 13:**
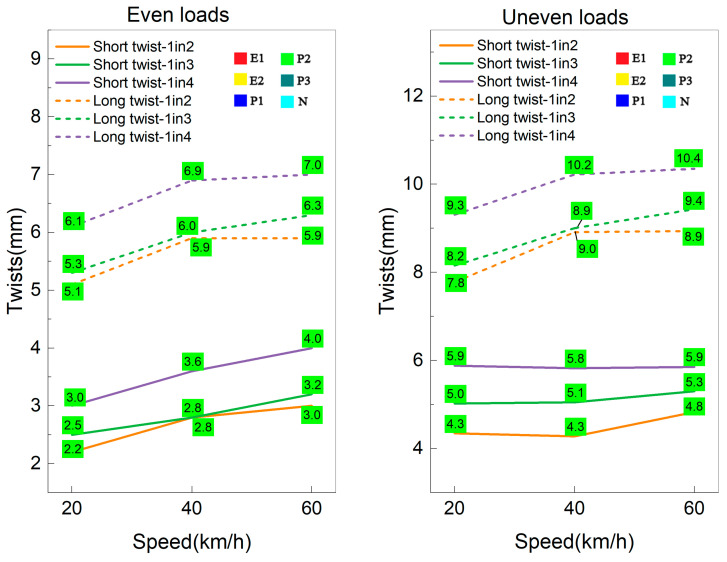
Dynamic twists of 1in2, 1in3 and 1in4 interspersed tracks under small-scale loss support and different load conditions.

**Figure 14:**
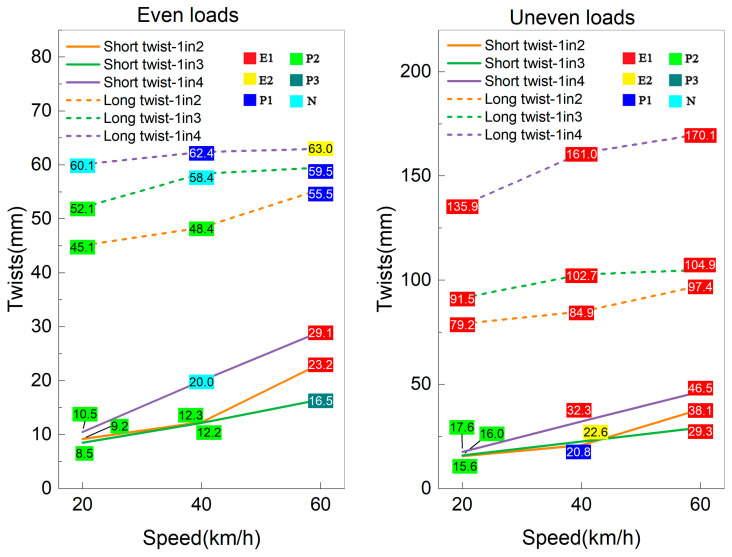
Dynamic twists of 1in2, 1in3 and 1in4 interspersed tracks under large-scale loss support and different load conditions.

The dynamic twists for 1in2, 1in3 and 1in4 interspersed tracks exposed to small-scale support loss conditions under even and uneven loads are illustrated in Figure 13. Even though the uneven loads induce much greater short and long twists than even loads in interspersed tracks, the small-scale support loss and uneven loads will not result in any twist problems for 1in2, 1in3 and 1in4 interspersed ballasted tracks at a speed less than 60 km/h. It is safe to operate a train with less than 60 km/h on an interspersed track in all conditions. Figure 14 shows the dynamic twists for all interspersed tracks exposed to large-scale support loss conditions. The uneven loads cause a boost in short twists and long twists at the same load and train speed, resulting in the track’s long twists exceeding the E1 limits. Immediate maintenance needed to be addressed to improve the track quality. Even though it is relatively safe for interspersed tracks exposed to large-scale loss support to operate under 60 km/h with even loads, the railway lines should be shut down if robust windy weather happens. As for the full-scale support loss condition, past research had already pointed out that 20 km/h would lead to an E1 situation with even loads [16]. The uneven moving loads induced by lateral wind loads and dynamic loads can cause much higher twists and accelerate the track deterioration, leading to train derailment. Thus, the railway lines are suggested to stop all operating trains until the track support state has been appropriately improved. It can be concluded from Figure 12 to Figure 14 that the interspersed track with higher concrete sleeper placement density (e.g., 1in2 track) performs better than lower concrete sleeper placement density tracks (e.g., 1in3 and 1in4 tracks); the uneven axle loads can amplify the dynamic response of track system and sometimes may result in more than double track twist values.

**Figure 15:**
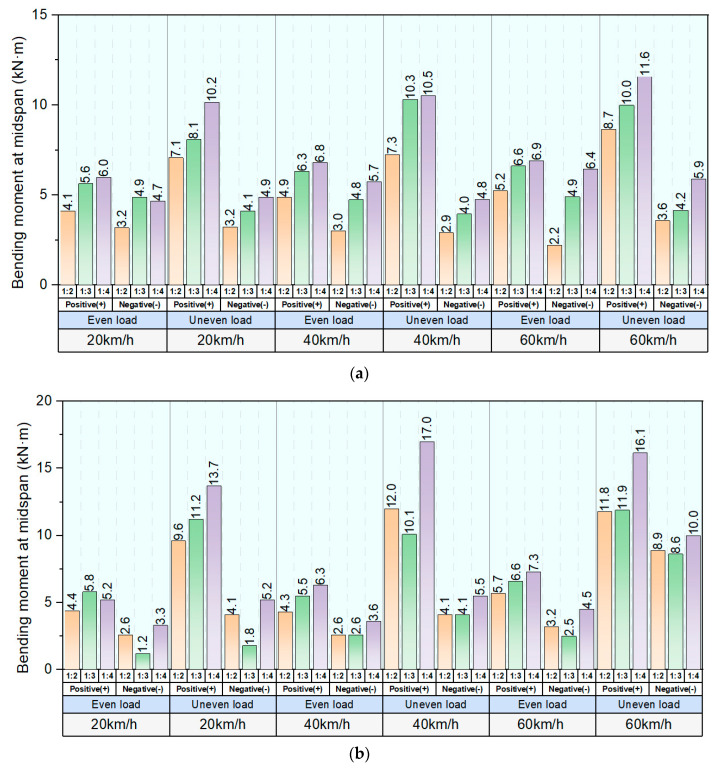

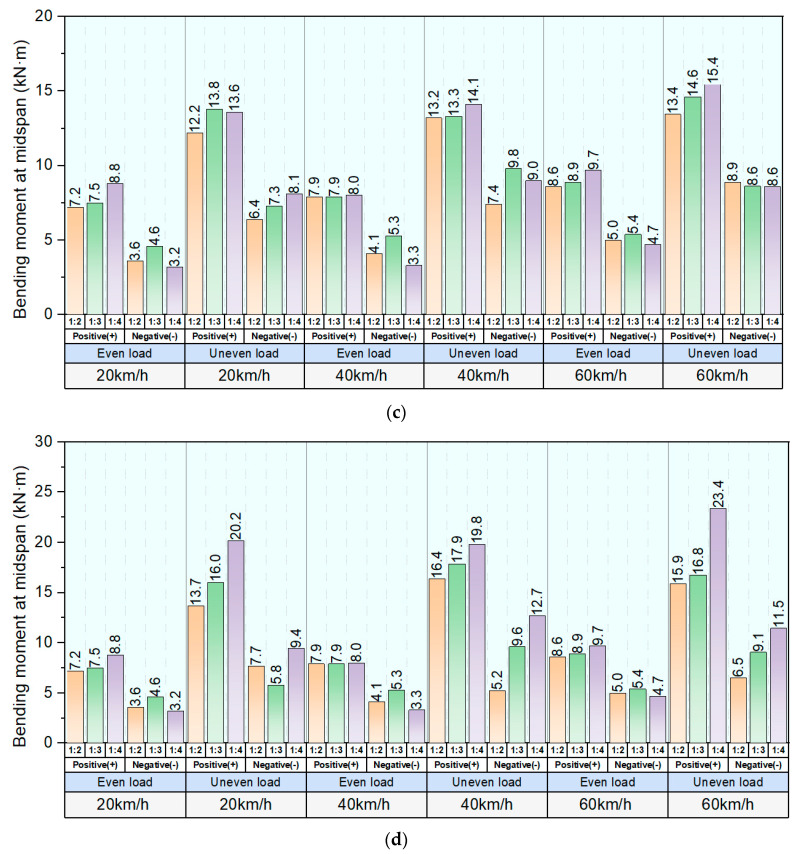
Maximum positive and negative bending moments of 1:2, 1:3 and 1:4 interspersed tracks at different track supporting states. (**a**) Maximum bending moments of interspersed tracks at normal support state, (**b**) Maximum bending moments of interspersed tracks at small–scale support loss state, (**c**) Maximum bending moments of interspersed tracks at large–scale support loss state and (**d**) Maximum bending moments of interspersed tracks at full–scale support loss state.

Figure 15a–d presents the maximum positive and negative bending moments at the midspan of interspersed tracks under different track supporting states and moving load conditions. The 1:2 interspersed track has the lowest average bending moments compared with the 1:3 and 1:4 interspersed tracks. The 1:2 track outperforms the 1:3 and 1:4 interspersed tracks in distributing flexural force under all track support states and load conditions. The uneven moving loads can induce higher positive and negative bending moments and increment the difference between positive and negative bending moment values. When exposed to the large-scale support loss condition, 1in2, 1in3, and 1in4 interspersed tracks have similar flexural responses at train speeds below 60 km/h.

### 3.2. Sensor Placement Strategies

From track responses obtained from FEM simulations, we can conclude that the sleeper support condition highly affects the dynamic performance of the interspersed railway track system. When the unsupported sleeper condition occurs with uneven axle loads, the dynamic responses can easily exceed the standard requirement’s safety limit. In order to ensure the safety of the interspersed railway lines, some measurements should be taken for the accurate early-age diagnosis and detection of the track defect. For example, the sleeper support conditions can be assessed and predicted using machine learning technologies based on the acceleration data of track components [57] and the rail displacement from digital video records [36]. Ground penetrating radar (GPR) can also reflect the ballast layer condition and the void zone [58], as well as on the bridge ends for digital twin based monitoring [59,60]. However, monitoring the sleeper support conditions on the ballasted railway line is challenging. Therefore, it is recommended to monitor the weak zones of railway lines, such as rail joints and turnouts, where support defects are prone. The acceleration sensors can be installed on the middle of sleepers and rails at an interval of a sleeper. Then, based on the acceleration data, the sleeper support condition and the scale of support loss can be predicted. After the sleeper support conditions are determined, the weather data from the Met office can be used as supplementary data to help determine the train speed limits for the day. However, only the axle load of 10 t is considered in this paper. More simulations should be studied for interspersed railway lines of different axle loads. In addition, the crosswind effect is simplified to uneven moving load while the axle loads are inconsistent. This also should be improved in the next step of the study. Knowing the sleeper support and weather conditions can help the railway operation’s safety.

## 4. Conclusions

In this paper, a set of interspersed track models have been built to study the vulnerability of the interspersed tracks exposed to heavy rain (flood) and crosswind conditions. The nonlinear finite analyses of interspersed ballasted tracks with different concrete sleeper placement densities (1in2, 1in3 and 1in4) have been performed. The dynamic twists of the tracks are compared with the Australian standard to assess the track defect level. The bending moments of interspersed tracks at midspan are compared to reveal the track flexural performance. The novelty of the research includes the adoption of tensionless supports, the consideration of ballast washaway caused by heavy rain (flood) and the unequal axle loads induced by crosswind loads. It should be noted that the current work simplifies the dynamic loads caused by the wheel–rail interaction and the lateral stability of the track system exposed to crosswind loads. In the future, more realistic loads will be investigated. This study would help railway engineers develop proper solutions towards extreme weather conditions. Based on the results and discussion, the following conclusions are given:

- Interspersed methods can improve the performance of existing conventional timber ballasted tracks. The interspersed track with higher concrete sleeper placement density performs better in resisting twist and flexural forces than lower concrete sleeper placement density. For example, the 1in2 interspersed track has better dynamic resistance to moving train loads than 1in3, 1in4 and timber ballasted tracks.
- All interspersed tracks (1in2, 1in3 and 1in4) at small-scale loss track support states can operate under 60 km/h without twist issues.
- The uneven axle loads caused by lateral wind loads and track defects can induce great twists and bending moments, lowering the track defect level and endangering safety. When strong winds accompany the heavy rain weather, the interspersed railway lines are suggested to be shut down.

## Figures and Tables

**Table 1:** Engineering parameters in the model.

Parameters	Value	Unit	Remarks
Track length	10.8	M	standard gauge is 1.435 m. 1.5 m is distance between wheel loads.
Load distance	1.5	M	
Rail modulus	200	GPa	
Rail Poisson’s ratio	0.25	-	
Rail density	7.85	g/mm^3^	
Rail-pad stiffness	17	MN/mm	
Concrete modulus	34.45	Gpa	
Concrete density	2.74	g/mm^3^	
Timber modulus	12.3	Gpa	
Timber density	1.25	g/mm^3^	
Ballast stiffness	17	MN/mm	

**Table 2:** Twist limits for ballasted track taken from Australian TMC 203 Track Inspection [55]. Note: N is normal condition, P is priority condition, E is emergency condition.

Track Geometry			Track Speed (Normal/Passenger) km/h					
Wide Gauge	Tight Gauge	Short Twist	20/20	40/40	60/60	80/90	100/120	115/160
<21	<10	<12	N	N	N	N	N	N
21–22	10	12–13	N	N	N	N	P3	P2
23–36	11–12	14–15	N	N	N	P3	P2	P1
27–28	13–14	16	N	N	P3	P2	P1	E2
29–30	15–16	17–18	N	P3	P2	P1	E2	E2
31–32	17	19–20	P2	P2	P1	E2	E2	E2
33–34	18	21–22	P1	P1	E2	E2	E2	E1
35–37	19–20	23	E2	E2	E2	E2	E1	E1
>37	>20	>23	E1	E1	E1	E1	E1	E1
Long Twist								
Not in Transition		Transition	20/20	40/40	60/60	80/90	100/120	115/160
<31		<34	N	N	N	N	N	N
31–35		34–38	N	N	N	N	P3	P2
36–40		39–43	N	N	N	P3	P2	P1
41–46		44–49	N	N	P3	P2	P1	E2
47–52		50–55	N	P3	P2	P1	E2	E2
53–59		56–62	P2	P2	P1	E2	E2	E2
60–64		63–66	P1	P1	E2	E2	E2	E1
65–70		66–72	E2	E2	E2	E2	E1	E1
>70		>72	E1	E1	E1	E1	E1	E1

**Table 3:** Repair requirements for six standard defect categories.

Response Category	Inspect and Verify Response	Action
Emergency 1 (E1)	Prior to passage of next train	Prior to passage of next train
Emergency 2 (E2)	Within 2 hours or before the next train, whichever is the greater	Within 24 hours
Priority 1 (P1)	Within 24 hours	Within 7 days
Priority 2 (P2)	Within 7 days	Within 28 days
Priority 3 (P3)	Within 28 days	Program for repair
Normal (N)	Nil	Routine inspection

## Data Availability

Data available on request from the authors.
